# A comparative analysis of surgically excised hereditary and sporadic pheochromocytomas: Insights from a single‐center experience

**DOI:** 10.1002/kjm2.12836

**Published:** 2024-05-15

**Authors:** Narin Nasiroglu Imga, Muzaffer Serdar Deniz, Belma Ozlem Tural Balsak, Yilmaz Aslan, Altug Tuncel, Dilek Berker

**Affiliations:** ^1^ Department of Endocrinology and Metabolism University of Health Science, Ankara City Hospital Ankara Turkey; ^2^ Department of Urology University of Health Science, Ankara City Hospital Ankara Turkey

**Keywords:** hereditary, pheochromocytoma, sporadic

## Abstract

Pheochromocytoma is a tumor that usually originating from adrenal medullary chromaffin cells and producing one or more catecholamines, can manifest as hereditary or sporadic. While the majority pheochromocytomas are sporadic, hereditary forms are often associated with genetic syndromes such as von Hippel–Lindau, multiple endocrine neoplasia type 2, and neurofibromatosis type 1. This study aims to analyze data from our series of surgically excited pheochromocytoma patients and compare the characteristics between hereditary and sporadic cases. We retrospectively evaluated 33 diagnosed pheochromocytoma patients, documenting clinical features, surgical complications, and tumor characteristics in both hereditary and sporadic cases. Among the patients, 21% (7 individuals) had hereditary pheochromocytoma, while 79% (26 individuals) had sporadic cases. During diagnosis, hereditary pheochromocytoma patients exhibited a significantly lower mean age compared to the sporadic group (26.4 ± 9.9 years vs. 50.4 ± 14.0 years; *p* < 0.001). The maximum tumor size was also lower in hereditary cases compared to sporadic cases (*p* = 0.004). Adrenal tumor localization analysis showed that 63.6% were right‐sided, 24.2% were left‐sided, and 12.1% were bilateral. Laboratory analysis revealed significantly higher urinary norepinephrine levels in hereditary pheochromocytoma patients (*p* = 0.021). Our findings suggest that hereditary pheochromocytoma cases are characterized by a younger age at diagnosis, smaller tumor size, and a higher prevalence of multiple bilateral adrenal adenomas. We recommend genetic testing for all pheochromocytoma patients, particularly those with early‐onset disease and bilateral adrenal tumors.

## 1 INTRODUCTION

Pheochromocytoma, a rare disease derived from neural crest chromaffin cell tumors, can secrete catecholamines into circulation and is often discovered incidentally. Its estimated occurrence in approximately 0.66 cases per 100,000 individuals per year.^1^ Hypertension in pheochromocytoma patients can be persistent or paroxysmal, with approximately 5%–15% being normotensive.^2^ When adrenal tumors present as adrenal incidentalomas, the prevalence of pheochromocytoma is about 1%–5%.^3^ Diagnosis is typically based on the symptomatic patient's history, with clinical presentation varying. Paroxysmal episodes of hypertension due to overproduction and extreme secretion of catecholamines are common, forming the classic triad of episodic tachycardia, headache, and sweating. Both sympathetic and parasympathetic pheochromocytomas may exhibit paroxysmal or persistent hypertension, headache, palpitation, syncope, anxiety, hyperglycemia, and sweating as the most common symptoms.^4^ Additionally, symptoms such as orthostatic hypotension, pallor, nausea, vomiting, weight loss, constipation, abdominal pain, flushing, weakness, fever, tremors, acute anxiety, and panic attacks may occur.^5^

Biochemical screening involves measuring plasma free catecholamines or urine‐fractionated catecholamines.^6^ While not widely available, plasma methoxytyramine testing may be useful in suspected metastatic disease cases.^7^ Circulating chromogranin A measurement in silent phenotypes may be a useful diagnostic marker, but there are many factors leading to false positive results.^8^ Following the laboratory diagnosis, tumor localization is recommended. Imaging methods include magnetic resonance imaging (MRI) or computed tomography (CT). Unenhanced CT scans showing adenoma HU > 10 in about 99.5% of cases.^9^ Radionuclide functional imaging methods, such as Gallium‐68‐DOTATATE positron emission tomography/computed tomography, have high sensitivity but are not widely available in most centers.^10^

Pheochromocytomas exhibit varying behavioral characteristics, with most being benign, although rare cases can be aggressive with a risk of metastasis. While predominantly sporadic, they can be related to hereditary syndromes like multiple endocrine neoplasia type 2A (MEN2A), MEN2B, von Hippel–Lindau (VHL) disease, and neurofibromatosis type 1 (NF‐1). Over 20 susceptibility pheochromocytoma genes have been identified genetically.^11^ Clinical genetic tests are advisable for all pheochromocytoma patients. Sporadic cases are typically detected in the 4th to 5th decade of life, while hereditary cases manifest at younger ages.^12^ This study aims to evaluate our diagnosed and surgically excised pheochromocytoma patient series, comparing the characteristics of hereditary and sporadic pheochromocytoma patients.

## 2 MATERIALS AND METHODS

We conducted a retrospective analysis of 33 surgically excised pheochromocytoma cases at our center from January 2008 to January 2019, with approval from the local ethics committee (ANEAH‐E‐18‐2050). Medical records, including laboratory and pathology results, preoperative and postoperative notes, and adrenalectomy operation details, were reviewed. General characteristics, such as age, gender, systolic blood pressure (SBP), diastolic blood pressure (DBP), and body mass index (BMI), were recorded, with BMI calculated by weight divided by height squared in meters. Patient data encompassed symptoms at diagnosis, family history, physical examination findings, radiological features, surgical complications, pathology results, and tumor characteristics. Diagnosis of VHL syndrome, MEN2A, and NF‐1 relied on family history, disease characteristics, and genetic confirmation. Sporadic pheochromocytoma was considered in the absence of clinical features and genetic mutations. Biochemical testing involved 24‐h urine fractionated metanephrines (metanephrine and normetanephrine) and catecholamines (epinephrine, norepinephrine, and dopamine) for diagnosis. Levels of at least two‐fold above the upper limit of the normal reference range indicated pheochromocytomas. Liquid chromatography with mass spectrometric method determined 24‐h urine fractionated metanephrines (normal range 50–250 μg/24 h), normetanephrine (15–80 μg/24 h), epinephrine (0–20 μg/24 h), and norepinephrine (100–500 μg/24 h). Plasma chromogranin A test, with a cutoff value of 92 μg/L, was performed for diagnosis in patients with normal 24‐h urinary catecholamine levels.

### 2.1 Statistical analysis

Statistical analysis was performed using the Statistical Package for Social Sciences v. 15 (SPSS, Chicago, IL). Categorical variables were represented by numbers or percentages. Normally distributed continuous variables were expressed as mean ± standard deviations. The independent *t*‐test was applied for normally distributed variables, and the Mann–Whitney *U* test was used for non‐normally distributed variables. Categorical variables were compared through the chi‐square test and Fisher's exact test. The distribution of patients was assessed using the histogram and the Kolmogorov–Smirnov tests. A *p*‐value <0.05 was considered statistically significant.

## 3 RESULTS

In this study, we performed an analysis on 33 patients diagnosed with pheochromocytoma. Table 1 provides characteristics and comparisons between hereditary and sporadic cases. Out of the total, 7/33 (21%) patients were diagnosed with hereditary pheochromocytoma, featuring a significantly lower mean age compared to the sporadic group (26.4 ± 9.9 vs. 50.4 ± 14.0; *p* < 0.001). There was no substantial difference in gender distribution (*p* = 0.413), and neither did BMI, SBP, nor DBP levels show significant variations (*p* > 0.05). The hereditary cases exhibited a diminished maximum tumor size (30.1 ± 16.5 mm vs. 56.2 ± 20.1 mm; *p* = 0.004). All bilateral adrenal adenomas were exclusively observed in hereditary cases (*p* < 0.001).

**TABLE 1:** General characteristics of all patients and comparison of hereditary and sporadic pheochromocytoma.

	All patients (*n* = 33)	Hereditary Pheo (*n* = 7)	Sporadic Pheo (*n* = 26)	*p* ^a^ value
Age (year)	49.3 ± 16.8	26.4 ± 9.9	50.4 ± 14.0	<0.001
Gender *n*, (%)				
Female	18 (54.5%)	5 (27.8%)	13 (72.2%)	0.413
Male	15 (45.5%)	2 (13.3%)	13 (86.7%)	
BMI (kg/m^2^)	29.6 ± 4.1	31.1 ± 3.7	29.3 ± 4.2	0.440
SBP (mmHg)	178.2 ± 16.1	177.5 ± 12.5	178.4 ± 17.1	0.920
DBP (mmHg)	105.6 ± 10.3	107.5 ± 9.6	105.3 ± 10.7	0.704
Radiologic tumor size (mm)	49.3 ± 16.8	39.4 ± 15.7	51.9 ± 16.4	0.080
Pathologic tumor size (mm)	50.7 ± 22.0	30.1 ± 16.5	56.2 ± 20.1	0.004
Tumor localization *n*, (%)				<0.001
Right	21 (63.6%)	3 (9.1%)	18 (54.5%)	
Left	8 (24.2%)	‐	8 (24.2%)	
Bilateral	4 (12.1%)	4 (12.1%)	‐	

Abbreviations: BMI, body mass index; DBP, diastolic blood pressure; Pheo, pheochromocytoma; SBP, systolic blood pressure.

^a^ Hereditary Pheo versus sporadic Pheo.

**FIGURE 1:**
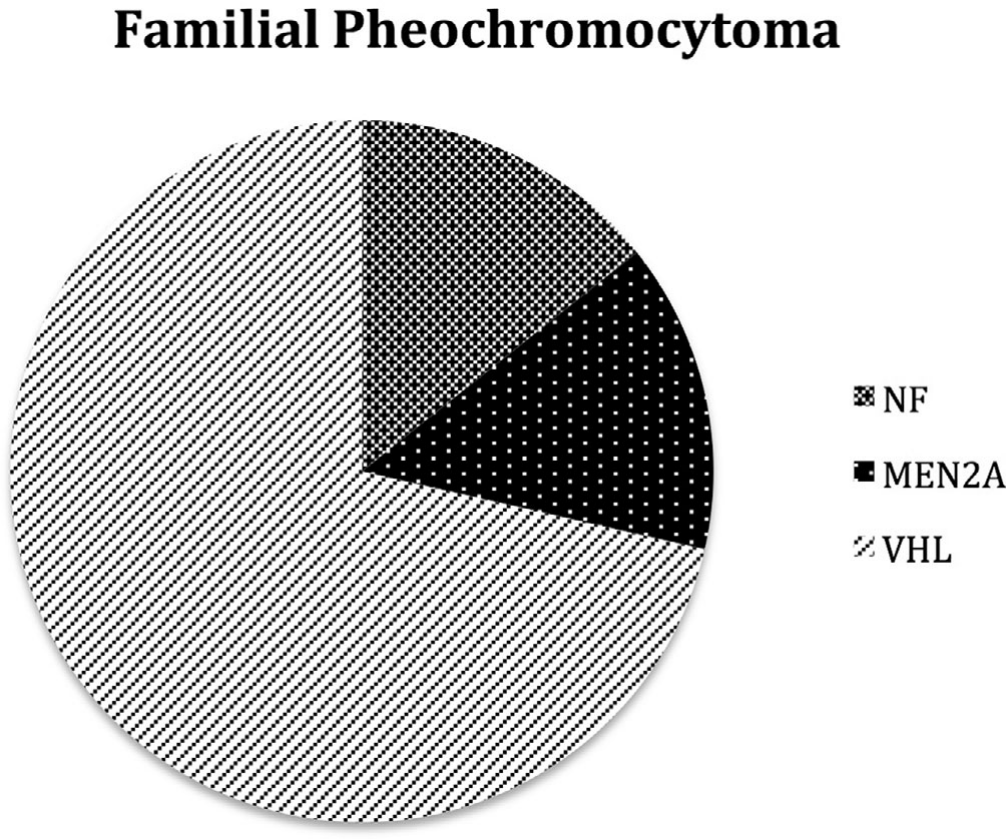
The distribution of patients with hereditary pheochromocytoma according to their diagnosis.

**FIGURE 2:**
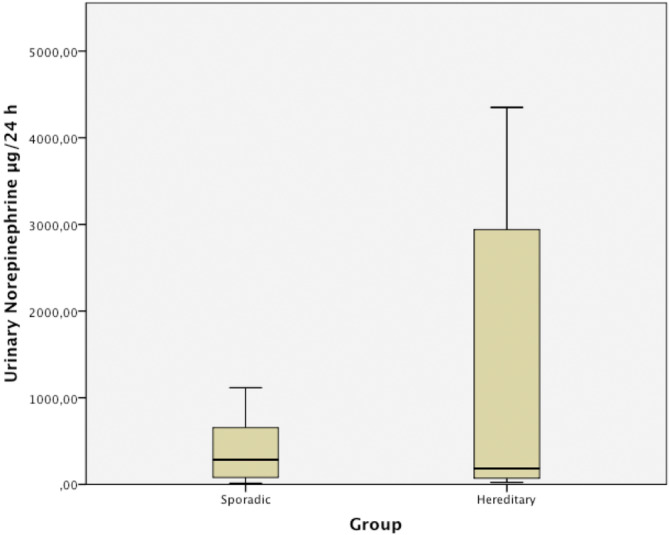
Urinary norepinephrine levels of sporadic and hereditary pheochromocytoma patients. Significantly (*p* = 0.021) different values in hereditary group than sporadic group.

Figure 1 elucidates the distribution of patients with hereditary pheochromocytoma. VHL emerged as the most frequently detected hereditary type (71.4%). Table 2 delineates clinical presentations, with hypertension (51.5%), headache (36.3%), and palpitations (33.3%) being prevalent. Asymptomatic patients constituted 33%. Table 3 shows the urinary excretion of catecholamine metabolites in all patients as well as compares the differences in hereditary and sporadic pheochromocytomas. The levels of 24‐h urinary epinephrine, metanephrine, and normetanephrine were found to be higher in hereditary pheochromocytoma than sporadic pheochromocytoma group but not statistically significant. However, 24‐h urinary norepinephrine excretion was found significantly higher in the hereditary pheochromocytoma group. Figure 2 further juxtaposes urinary norepinephrine levels between hereditary and sporadic pheochromocytoma patients (1513.8 ± 2009.7 vs. 394.8 ± 360.5; *p* = 0.021).

**TABLE 2:** Clinical features of 33 patients with pheochromocytoma at the time of diagnosis.

	*n* (%)
Hypertension	17 (51.5)
Flushing	4 (12.1)
Palpitations	11 (33.3)
Headache	12 (36.3)
Sweating	5 (15.1)
Abdominal pain	4 (12.1)
Anxiety	6 (18.2)
Fatigue	8 (24.2)
Weight loss	2 (6.1)
Asymptomatic	11 (33.3)

**TABLE 3:** Comparison of the urinary cathecolamines and metanephrines between hereditary and sporadic pheochromocytomas.

	All	Hereditary Pheo	Sporadic Pheo	*p* ^a^
Urinary epinephrine (μg/24 h)	123.6 ± 254.1	196.1 ± 418.1	105.6 ± 207.5	0.488
Urinary norepinephrine (μg/24 h)	618.6 ± 992.3	1513.8 ± 2009.7	394.8 ± 360.5	0.021
Urinary metanephrine (μg/24 h)	1282.7 ± 2224.1	1737.0 ± 3642.8	1163.1 ± 1816.5	0.619
Urinary normetanephrine (μg/24 h)	652.7 ± 708.7	940.4 ± 1265.5	577.0 ± 506.9	0.318
Urinary dopamine (μg/24 h)	371.6 ± 446.5	333.5 ± 247.7	381.7 ± 492.6	0.854

^a^ Hereditary pheochromocytoma versus sporadic pheochromocytoma.

Additionally, three sporadic cases with normal urinary metanephrine and catecholamine levels underwent chromogranin A tests. Intraoperative phentolamine requirement was observed in five (9%) patients, and postoperative persistent hypertension in seven (21%) patients.

## 4 DISCUSSION

Catecholamine‐secreting tumors, including pheochromocytomas, are seen rare. Pheochromocytomas can occur at any age but are most commonly found in the fourth to fifth decades of life, with no significant gender differences observed.^13^ In our patient series, the mean age among all patients was 49.3 ± 16.8 years, aligning with the literature that places sporadic pheochromocytomas predominantly in the 5th decade. Additionally, our analysis revealed no significant differences in gender distribution. While most pheochromocytomas are sporadic, approximately 25% are estimated to be hereditary.^14^ Notably, hereditary pheochromocytoma typically presents at younger ages and with smaller tumors compared to sporadic cases.^15^, ^16^ Our data support this, showing a significantly younger mean age for hereditary cases at the time of diagnosis (*p* < 0.001).

Pheochromocytomas are associated with a wide range of symptoms due to excessive catecholamine secretion. The classical symptom triad in pheochromocytoma patients includes headaches, sweating, and tachycardia. However, these symptoms are not present in the majority of cases.^17^ The most prevalent symptom is hypertension, which can be either sustained or paroxysmal.^18^ In our patient cohort, while the mean systolic and diastolic blood pressure values were high, they did not significantly differ between hereditary and sporadic pheochromocytoma groups. The most common symptoms observed were hypertension, followed by headaches and palpitations. Diabetes and prediabetes may signal a hyperadrenergic condition. A previous study noted a lower prevalence of BMI above 30 kg/m^2^ in patients diagnosed with pheochromocytoma.^19^ Our findings indicated higher BMIs in both hereditary and sporadic groups, with no significant difference between them. This trend might be reflective of the general prevalence of overweight individuals in our population.

Additionally, we observed smaller adenoma sizes in the hereditary group. This suggests that individuals with genetic predispositions may develop symptoms at earlier ages and form smaller tumors due to specific gene mutations. This finding underscores the importance of genetic testing, especially for young patients, for earlier diagnosis and intervention. Past researches have shown that hereditary pheochromocytomas tend to be multiple and bilateral,^20^, ^21^ which was consistent with our findings where all bilateral adrenal pheochromocytomas were hereditary. This supports the hypothesis that pheochromocytoma cases associated with specific genetic syndromes may be linked to a more aggressive disease course. Therefore, treatment strategies and follow‐up protocols for hereditary pheochromocytoma cases may need to be adjusted for disease course.

Understanding the genetic keystones of familial and hereditary pheochromocytomas holds significant clinical importance and can impact management strategies. Genetic testing plays a pivotal role in identifying at‐risk individuals, facilitating early diagnosis, and guiding surveillance protocols. This enables the customization of treatment strategies encompassing surgical intervention, pharmacotherapy, and monitoring protocols. Therefore, a better understanding of familial and hereditary pheochromocytomas holds promise for significant advancements in disease diagnosis and management. Further exploration of genetic mechanisms may contribute to improving clinical outcomes and advancing approaches.

Guidelines suggest that initial screening for pheochromocytomas and paragangliomas should include measurements of plasma‐free metanephrines or urine‐fractionated metanephrines. Our study revealed elevated urinary metanephrine levels, although these were not significantly different between hereditary and sporadic cases. It is known that pheochromocytomas associated with MEN 2 express the phenylethanolamine‐N‐methyltransferase (PNMT) enzyme, which converts norepinephrine to epinephrine. However, VHL‐associated pheochromocytomas do not typically express PNMT.^22^ This distinction was evident in our study, where we observed elevated urinary norepinephrine levels predominantly in hereditary cases, likely due to the higher prevalence of VHL. Postoperative urinary metanephrine levels, measured 6 weeks after surgery, were found to be within the normal range for all patients. Genetic counseling and testing are crucial for patients diagnosed with pheochromocytomas.^23^ In our study, genetic counseling was provided to patients with hereditary pheochromocytomas, and the genetic results confirmed the presence of vHL, NF‐1, and MEN2A. Chromogranin A, an essential protein marker for pheochromocytomas, should be assayed preoperatively in patients with normal preoperative plasma or urinary levels of metanephrine and normetanephrine.^6^ We conducted chromogranin A tests in three cases where urinary metanephrine and catecholamine levels were normal. All these cases were identified as sporadic pheochromocytomas. Elevated serum chromogranin A levels facilitated the diagnosis of pheochromocytoma in these patients and informed our caution regarding potential hypertension attacks during surgery.

Our study has some limitations. Firstly, due to the rarity of pheochromocytoma, the sample size was small, particularly impacting the comparison of clinical, laboratory, and radiological features. Secondly, our center only evaluated urinary catecholamines, as plasma catecholamines were not studied. Optimal diagnostic sensitivity requires that the plasma‐free metanephrine test be conducted on blood samples collected after 30 min of supine rest and following an overnight fast. However, in high‐capacity hospitals with heavy patient loads, ensuring these conditions can be challenging. Thirdly, our study's retrospective nature limited certain aspects; for instance, the plasma methoxytyramine test was not performed due to its recent introduction in our laboratory.

In conclusion, our study provides valuable insights into the genetic and clinical characteristics of patients diagnosed with pheochromocytomas. We observed younger patients with smaller‐sized adrenal tumors tend to be hereditary pheochromocytomas. Additionally, hereditary pheochromocytomas were more likely to manifest as bilateral adrenal tumors and were associated with higher 24‐h urinary norepinephrine levels. Identifying differences between hereditary and sporadic cases may contribute to a better understanding of the pathophysiology of the disease and treatment strategies. Future research, conducted on larger sample groups, can further contribute to a better understanding of these differences and inform clinical practice.

## CONFLICT OF INTEREST STATEMENT

The authors declare no conflict of interest.
